# Structural and biochemical characterization of an RNA/DNA binding motif in the N-terminal domain of RecQ4 helicases

**DOI:** 10.1038/srep21501

**Published:** 2016-02-18

**Authors:** Francesca Marino, Aditya Mojumdar, Chiara Zucchelli, Amit Bhardwaj, Emanuele Buratti, Alessandro Vindigni, Giovanna Musco, Silvia Onesti

**Affiliations:** 1 Structural Biology Laboratory, Elettra-Sincrotrone Trieste S.C.p.A., Trieste, Italy; 2 Dipartimento di Scienze della Vita, Università degli Studi di Trieste, Italy; 3 Biomolecular NMR Unit, Division of Genetics and Cell Biology, S. Raffaele Scientific Institute, Milano, Italy; 4 International Centre for Genetic Engineering and Biotechnology (ICGEB), Trieste, Italy; 5 Department of Biochemistry and Molecular Biology, Saint Louis University School of Medicine, St. Louis, Missouri, USA

## Abstract

The RecQ4 helicase belongs to the ubiquitous RecQ family but its exact role in the cell is not completely understood. In addition to the helicase domain, RecQ4 has a unique N-terminal part that is essential for viability and is constituted by a region homologous to the yeast Sld2 replication initiation factor, followed by a cysteine-rich region, predicted to fold as a Zn knuckle. We carried out a structural and biochemical analysis of both the human and *Xenopus laevis* RecQ4 cysteine-rich regions, and showed by NMR spectroscopy that the *Xenopus* fragment indeed assumes the canonical Zn knuckle fold, whereas the human sequence remains unstructured, consistent with the mutation of one of the Zn ligands. Both the human and *Xenopus* Zn knuckles bind to a variety of nucleic acid substrates, with a mild preference for RNA. We also investigated the effect of a segment located upstream the Zn knuckle that is highly conserved and rich in positively charged and aromatic residues, partially overlapping with the C-terminus of the Sld2-like domain. In both the human and *Xenopus* proteins, the presence of this region strongly enhances binding to nucleic acids. These results reveal novel possible roles of RecQ4 in DNA replication and genome stability.

RecQ helicases are ubiquitous enzymes involved in the maintenance of genome stability, acting in DNA repair, replication, and recombination. Yeasts and bacteria express only one or two members of this family, while five RecQ helicases are found in humans, namely RecQ1, Blm, Wrn, RecQ4 and RecQ5. Germ line mutations in genes coding for three of five human RecQ enzymes are associated with autosomal recessive disorders, characterized by increased genome instability, premature aging, and cancer predisposition. While sharing some clinical outcomes, patients affected by RecQ-related syndromes show different phenotypes, underlying a specific role of each RecQ enzyme within the cell^1^. In particular, mutations in *BLM* gene cause the Bloom’s syndrome and in *WRN* gene the Werner’s syndrome, while mutations in *RECQL4* gene are associated with three diseases, the Rothmund-Thomson (RTS), RAPADILINO and Baller-Gerold syndromes. Mutations in *RECQL4* gene are present in two third of clinically diagnosed RTS patients and this subset of patients is at a high risk to develop osteosarcoma^2^. Moreover, RecQ4 is overexpressed in several cancer types and its role in carcinogenesis of prostate and breast cancer has been recently established^3^,^4^.

The human RecQ4 protein is 1208 amino acids long and comprises the conserved helicase domain of the SF2 helicase family, followed by a putative RecQ C-terminal domain (RQC, Fig. 1A)^5^. The N-terminal region includes a region of homology to yeast Sld2, an essential factor in yeast DNA replication. The presence of an Sld2-like domain is a feature unique to RecQ4 proteins within the RecQ family^6^. RecQ4 has indeed a prominent role in the initiation of eukaryotic DNA replication^6^,^7^,^8^. For example, RecQ4 appears to be a limiting factor for replication initiation in *Xenopus laevis*, together with DNA replication factors Cut5, Drf1 and Treslin^9^. The importance of RecQ4 in DNA replication is in agreement with the fact that deletion of the N-terminal region of RecQ4 causes lethality in mice models^10^. Consistently, most of the mutations observed in patients are localized in the helicase or RQC domains, suggesting that the first 450 amino-acid residues are very important for viability^2^,^11^. RecQ4 is also involved in DNA repair, telomere maintenance and mitochondrial DNA protection^1^,^12^,^13^.

Despite its importance, a full biochemical characterization of RecQ4 is still missing. Initial reports suggested that the protein was inactive^14^,^15^, whereas subsequent studies detected a weak but reproducible helicase activity^16^,^17^. A recent *in silico* analysis of RecQ4 proteins^5^ has highlighted two important features in their N-terminal region: a second region of homology with Sld2^5^,^18^, corresponding to the C-terminus of the yeast replication factor (in addition to the N-terminal 150 amino-acid residues originally described by Sangrithi and colleagues^6^), and a cysteine-rich region classified as “retrovirus Zn finger like” or “Zn knuckle”, located between the Sld2 homology region and the helicase domain (Fig. 1C). The newly identified region of homology to Sld2, located immediately upstream the Zn knuckle, is highly conserved among species and includes numerous positively charged and aromatic residues; interestingly, a known RTS mutation involves one of these aromatic residues (Trp383^10^).

Zn knuckles are short cysteine-rich sequences wrapping around a Zn^2+^ ion, often present in multiple copies in nucleocapsid proteins of RNA retroviruses and in eukaryotic gene regulators. The best studied are the C-terminal Zn motifs of the HIV-1 nucleocapsid protein NCp7, where Zn^2+^ binding is necessary for most protein functions^19^, and the RNA binding protein Lin-28, that contains two Zn knuckles involved in binding and processing members of the pre-let-7 family of miRNAs^20^,^21^. Within the RecQ4 paralogues, the Zn knuckle motif is well-conserved albeit with occasional variants of the canonical Zn site: for example the human sequence has the second cysteine substituted by an asparagine (CNHC) while the *Xenopus laevis* region contains the canonical Zn ligands: CCHC (Fig. 1C).

Structural information on RecQ4 proteins is limited to the first 54 amino acids at the very N-terminus (within the Sld2 homology region) that form a helical bundle resembling a homeodomain^22^. Two biochemical characterizations of fragments of the N-terminal domain have been recently published, but neither include the potential Zn knuckle region^23^,^24^.

In this work, we have investigated by CD and NMR spectroscopy the human and frog RecQ4 Zn knuckles and characterized their nucleic acid binding properties. We have determined the three-dimensional structure of the canonical *X. laevis* Zn knuckle by NMR. We also analyzed the effect of the newly identified Sld2 homology region on the biochemical properties of the Zn-binding domains.

## Results

### RecQ4 N-terminus contains a Zn-knuckle motif

**Figure 1:**
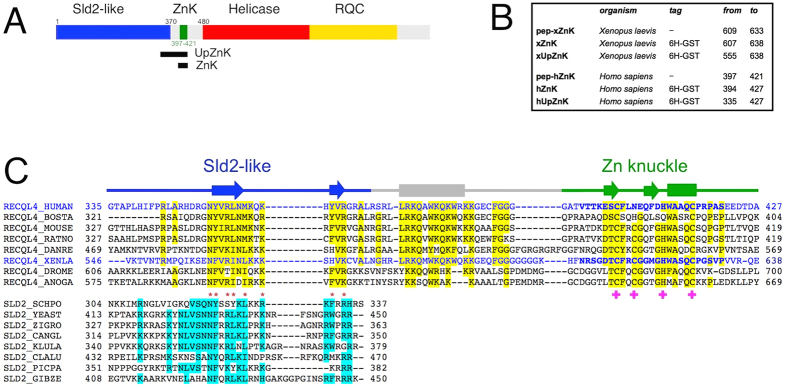
Analysis of RecQ4 N-terminal region. (**A**) Structural organization of human RecQ4: the extended Sld2 homology region (blue), the Zn knuckle (green), the helicase core (red) and the RecQ C-terminal domain (RQC, in yellow). The two black rectangles indicate the regions encompassed by the protein constructs made for this study (ZnK and UpZnK). (**B**) Details of the synthesised peptides (pep-xZnK, pep-hZnK) and recombinant protein fragments (xZnK, hZnK, xUpZnK, hUpZnK) analysed in the present study. (**C**) Sequence alignment between RecQ4 and the C-terminus of Sld2 proteins. The human and *Xenopus* sequences are coloured in blue and the residues of the two synthetic peptides pep-hZnK and pep-xZnK are in bold. Residues that are conserved in 6/8 sequences are highlighted in yellow and cyan in RecQ4 and Sld2 proteins, respectively. Residues such as R/K/H, D/E, S/P/T/C, A/G, Y/F/W, I/L/V/M and Q/N, are classified as conserved. Residues conserved across RecQ4 and Sld2 are indicated by red asterisks. Amino acids involved in Zn^2+^ coordination are indicated by pink crosses. For the Zn knuckle the secondary structure elements (α-helices as rods and β-strands as arrows) are based on the *Xenopus* NMR structure (PDB ID: 2MPJ), otherwise they are based on the PsiPred prediction (http://bioinf.cs.ucl.ac.uk/psipred/).

**Figure 2:**
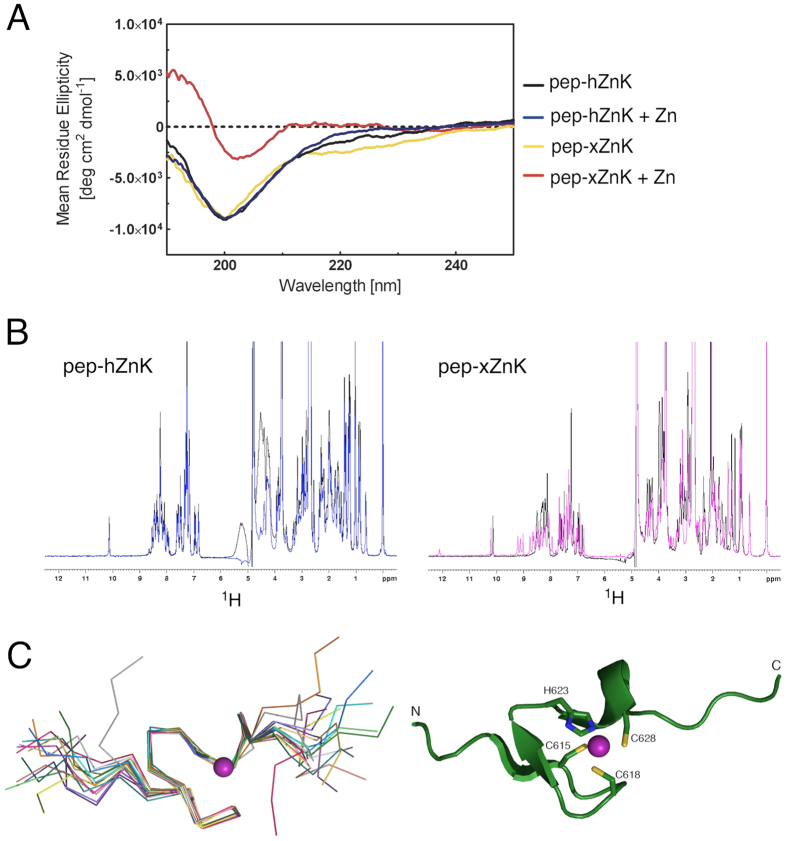
Spectroscopic studies of the peptides corresponding to the human and *Xenopus* Zn knuckles (pep-hZnK and pep-xZnK). (**A**) The CD spectra of the two peptides are characteristic of unfolded proteins (black and yellow, respectively). The addition of Zn^2+^ in equimolar amount, triggers the formation of secondary structures for the *Xenopus* peptide but not for the human one. (**B**) Left: 1D ^1^H NMR spectra of pep-hZnK in absence (black) and presence (blue) of Zn^2+^. Right: 1D ^1^H NMR spectra of pep-xZnK in absence (black) and presence (pink) of Zn^2+^. (**C**) Left: NMR bundle of the best 15 structures for the *Xenopus* Zn knuckle peptide (pep-xZnK). Right: Cartoon representation of one representative NMR structure. The Zn^2+^ metal ion is in purple and residues involved in Zn^2+^ coordination are depicted as ball-and-sticks.

The presence of a cysteine-rich motif [(S/T)**C**(F/Y)x**C**Gxx**H**WAxQ**C**] in the N-terminal domain of RecQ4 strongly suggests the presence of a Zn knuckle, despite occasional variation in the canonical Zn ligands (Fig. 1B). In order to investigate whether this knuckle is an autonomous structural element and to verify whether the observed changes in the consensus site did affect the structure and/or function of the human protein, we chemically synthesized two 25 amino-acid long peptides corresponding to the human (residues 397 to 421, pep-hZnK) and *Xenopus* (residues 609 to 633, pep-xZnK) Zn knuckles (Fig. 1B). As mentioned above, the human sequence contains a CNHC Zn binding site, while in *Xenopus* RecQ4 the consensus CCHC is maintained. Both peptides were analyzed by CD spectroscopy and in absence of Zn^2+^ showed a spectrum characteristic of unfolded polypeptides. The addition of Zn ions causes changes to the spectrum of the *Xenopus* Zn knuckle, compatible with the presence of secondary structure, while the human peptide remained unchanged (Fig. 2A). The results were confirmed by acquiring NMR 1D ^1^H spectra for the two peptides in the presence or absence of Zn^2+^ in phosphate buffer (Fig. 2B), suggesting that while the *Xenopus* fragment folds upon Zn^2+^ addition, the human peptide remains unstructured. As phosphate salt might sequester zinc, we repeated the NMR experiment for the human peptide in HEPES without observing any folding (Supplementary Fig. S1 online). This is consistent with the notion that the human Zn knuckle has lower affinity to zinc due to the asparagine mutation.

To test whether Zn binding and folding may be triggered by the presence of a physiological substrate, we repeated the CD experiments in presence of ssDNA or ssRNA. However, we did not observe any change in the presence of nucleic acids. As a further check we collected the 1D NMR spectrum in the presence of ssDNA without detecting any folding (Supplementary Fig. S1 online).

### NMR structure of the *Xenopus laevis* RecQ4 Zn-knuckle

We determined the solution structure of *Xenopus laevis* RecQ4 Zn knuckle (pep-xZnK: Asn609-Pro633, Fig. 1B) by bidimensional homonuclear ^1^H NMR spectroscopy (Fig. 2C; structural statistics in Table 1). Upon Zn^2+^ addition the peptide folds into a small globular domain wrapping around one Zn^2+^ ion that is coordinated by three cysteines and one histidine (Cys615, Cys618, His623 and Cys628, Fig. 2C). The domain is well ordered between residues Thr614 and Cys628, with a RMSD of 0.243 Å over the backbone atoms. Typical i-i + 3 NOE cross-peaks such as Ala625_Hα_/Cys628_HN_ and Ala625_Hα_/Cys628_Hβ1/Hβ2_ indicate the presence of an α-helical turn comprising Ala625–Cys628. Long range NOE cross peaks (e.g. Gly620_HN_/Cys615_HN_, Gly622_HN_/Thr614_Hα_) reveal the presence of a short antiparallel β-sheet, formed by residues Asp613–Cys615 (β1) and Gly620–Gly622 (β2) (Supplementary Fig. S2 online). Of note, Trp624 and Phe616 are partially exposed to the solvent, suggesting a possible functional role for these residues.

A search for structural homologues of the *Xenopus* RecQ4 Zn knuckle using PDBefold^25^ finds a number of Zn knuckles, including the C-terminal Zn knuckle of the microtubule plus-end tracking protein CLIP-170 and a variety of similar folds in viral nucleocapsides and eukaryotic regulators (with Q-scores ranging from 0.67 to 0.43).

### Nucleic acid binding abilities of the Zn-knuckle domain

Many Zn knuckles are capable of binding a wide range of nucleic acid substrates (mainly RNA). Thus, we expressed in *E. coli* the human (hZnK) and *Xenopus* (xZnK) RecQ4 Zn knuckles fused to a N-terminal GST tag in order to evaluate their role in nucleic acid binding by electrophoretic mobility shift assays (EMSA). Given the putative role of RecQ4 in DNA replication we used DNA and RNA substrates resembling a replication fork, in addition to single and double-stranded substrates (Supplementary Table S1 online).

**Figure 3:**
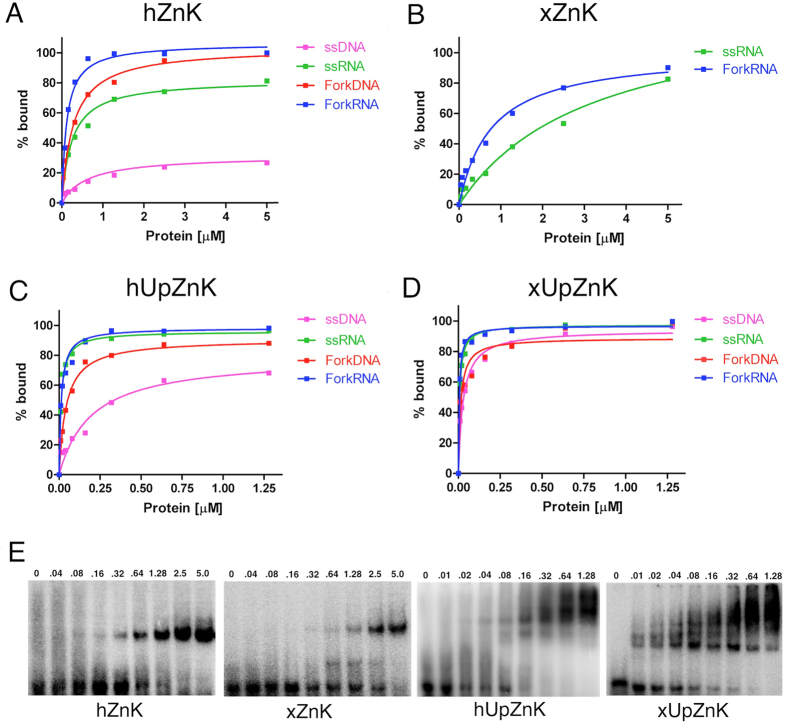
Quantitative EMSA analysis of various DNA/RNA complexes with the recombinant fragments of human and *Xenopus* RecQ4 corresponding to the Zn knuckle alone (hZnK and xZnK, panels (**A**,**B**) and the Zn knuckle with the upstream conserved region (hUpZnK and xUpZnK, panels (**C**,**D**). (**E**) Example of gel shift assays for the various protein fragments using a forked RNA substrate. The assay was carried out with increasing concentrations of proteins (0–5 μM for the hZnK and xZnK; 0–1.28 μM for the hUpZnK and xUpZnK). Recombinant proteins include a 6His-GST tag. Each experiment was repeated at least three times to plot the binding curves. Errors were very small: for the sake of clarity error bars are not shown on the plots.

The human Zn knuckle, that appears to be unstructured on the basis of our NMR analysis, showed weak binding to ssDNA (*K*_D _≈ 0.5 μM). However, it binds either ssRNA or fork substrates (DNA or RNA) with significantly higher affinity (Fig. 3A). The approximate *K*_D_ values were 270 nM for forkDNA, 220 nM for ssRNA and 130 nM for forkRNA.

The *Xenopus* Zn knuckle, that possesses an intact CCHC site, in the same conditions showed no detectable binding to either ssDNA or forkDNA substrates. However, it was able to weakly bind both ss and forked RNA with *K*_D_ of 1–3 μM (Fig. 3B). For both Zn knuckles, no binding was observed to double-stranded DNA substrates of 22 base-pairs equivalent to the annealed region of the fork substrates (data not shown).

We conclude that the RecQ4 Zn knuckle preferentially binds forked substrates with a preference for RNA, and that the human sequence is able to bind nucleic acids despite the lack of the canonical Zn-binding consensus. The presence of ZnCl_2_ in the binding buffer significantly enhances the binding of the human Zn knuckle to both DNA and RNA forked substrates (Supplementary Fig. S3 online), indicating that Zn^2+^ ions plays a relevant role in the interaction, and suggesting that the folding of the Zn knuckle is indeed important for the interaction.

### Role of the additional Sld2 homology region upstream the Zn-knuckle

A highly conserved region, partly similar to the C-terminus of the yeast Sld2 initiation factors, is present upstream the Zn knuckle and includes many positively charged and aromatic residues (Fig. 1B). We therefore decided to investigate the role of this region in modulating the nucleic acid binding properties of the Zn knuckle.

Two constructs containing residues 335 to 427 of the human sequence and residues 555 to 638 for the *Xenopus* sequence (therefore including both the Zn knuckle and the upstream region) were designed, and the corresponding proteins were purified, fused to a His-GST tag (hUpZnK and xUpZnK, respectively, Fig. 1B).

Both the human and *Xenopus* fragments bind single-stranded and fork substrates with affinities that are consistently higher than the Zn knuckles alone (Fig. 3C–E). The affinity constants for each complex are reported in Table 2. Contrary to what seen with the Zn knuckles alone, in the presence of the upstream region, the *Xenopus* protein binds more tightly all the substrates. Both proteins displayed a preference for RNA, with similar affinity for the single-stranded and forked RNA substrates. No binding to a 22 bp DNA duplex was observed (data not shown).

**Figure 4:**
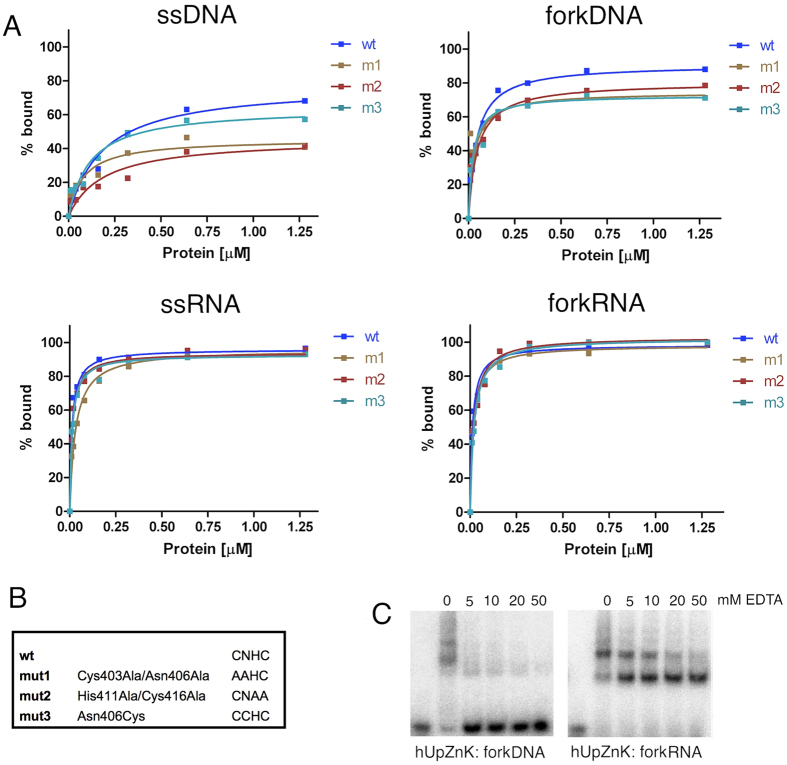
Role of the Zn ligands in nucleic acid binding. (**A**) Binding curves for wild type hUpZnk and mutants (m1: Cys403Ala/Asn406Ala; m2: His411Ala/Cys416Ala; m3: Asn406Cys) with ssDNA, forkDNA, ssRNA and forkRNA. Each experiment was repeated at least three times. (**B**) Summary of the site-directed mutants. (**C**) Binding of the hUpZnK fragment to forkDNA and forkRNA substrates, in the presence of increasing amount of EDTA. The presence of EDTA strongly affects binding to DNA substrates, while does not impair binding to RNA, although it changes the relative intensity of the shifted bands.

To further dissect the interaction hUpZnK was subjected to site-directed mutagenesis to target the Zn ligands. Two mutants were designed to disrupt Zn^2+^ binding (m1: Cys403Ala/Asn406Ala; m2: His411Ala/Cys416Ala) and one to restore the canonical CCHC binding site (m3: Asn406Cys, Fig. 4B). Alanine mutations weakened the binding of the protein to DNA substrates (ssDNA, fork DNA) whereas the affinity towards RNA substrates did not change dramatically (Fig. 4A). The mutation of the Asn to Cys, reconstituting a canonical Zn knuckle pattern, did not improve binding to any of the substrates; on the contrary it negatively affected binding to DNA substrates, similarly to the Zn ligands disrupting mutants.

We confirmed these results by carrying out nucleic acid binding experiments in the presence of increasing amounts of EDTA (Fig. 4C). EDTA strongly affects binding to forked DNA substrates, while it does not eliminate the interaction with RNA: it does however change the relative intensity of the shifted bands, suggesting that the Zn knuckle modulates the interaction with RNA.

## Discussion

Most of the RecQ4 mutations causing genetic syndromes are located in the second half of the protein. Indeed both knockout mice^10^ and *in vivo* studies^18^,^26^ indicate that the N-terminal region (up to residue 495) is required for viability and the initiation of DNA replication.

The N-terminal domain of RecQ4 is unique among the RecQ helicase and shares a weak but significant homology to the yeast factor Sld2, an essential factor in the initiation of DNA replication^5^,^6^. Whereas the first segment, including amino acids 1–54, has been biochemically and structurally characterized^22^ no data are available for the remaining of the N-terminal domain. The presence of low complexity regions in the sequence suggests that a large portion of the N-terminal domain is intrinsically unstructured, with only a few segments predicted to assume a defined secondary structure. Wedged between the Sld2-homology region and the canonical RecQ helicase domain is a cysteine-rich region that conforms to the pattern of a Zn knuckle domain. The region immediately upstream the Zn knuckles is also very well conserved within RecQ4 proteins, and comprises a stretch that is homologous to Sld2 proteins and one that is unique to RecQ4 helicases (Fig. 1B).

We carried out a structural and functional analysis to address the following questions: does the cysteine-rich region fold as a Zn knuckle?is the Zn knuckle involved in interactions with nucleic acids?does the Cys- >Asn mutant in the human sequence affect the structure or function of the protein?what is the role of the highly conserved upstream region?

We have produced two synthetic peptides corresponding to the Zn knuckles of human and *Xenopus* RecQ4, and found by CD and NMR spectroscopy that only the frog peptide (pep-xZnK), containing the canonical CCHC consensus, is able to fold upon Zn^2+^ addition (Fig. 2A,B). The human Zn knuckle (pep-hZnK) remained in an unfolded state under a number of buffer conditions. The addition of nucleic acid substrates did not cause any detectable change, suggesting that folding is not triggered by the presence of substrates (Supplementary Fig. S1 online).

We have determined the NMR structure of pep-xZnK (Fig. 2C) that conforms to the canonical Zn knuckle fold and is composed by a very short α-helix and an antiparallel β-sheet (ββα fold) surrounding a single Zn^2+^ ion coordinated by three cysteine and one histidine residues.

In order to study the nucleic acid binding abilities of RecQ4 human and *Xenopus* Zn knuckles by EMSA analysis, we have cloned the corresponding regions fused to a His-GST tag (hZnK and xZnK) for bacterial expression. Both the human and the *Xenopus* Zn knuckles show binding to RNA substrates, with a preference for forked structures (Fig. 3A,B). The human knuckle can also bind DNA, although with lower affinity.

There is an apparent discrepancy between the EMSA results, showing that the human Zn knuckle alone binds nucleic acid substrates and the spectroscopic analysis (by CD and NMR) that does not detect any folding upon addition of nucleic acid (Fig. 2 and Supplementary Fig. S1 online). Although there are numerous cases of unstructured proteins that bind nucleic acids, the sequence similarity between the human fragment and canonical Zn knuckles (such as the *Xenopus* one) would suggest that the human protein may assume a similar fold upon nucleic acid binding; presumably the population bound to the nucleic acid, and thus assuming a folded conformation, is too unstable and/or too small to be detectable by our spectroscopic analysis. Indeed the presence of Zn^2+^ ions in the buffer improves the affinity of the human fragments for both DNA and RNA (Supplementary Fig. S3 online), suggesting that the active form of the protein does indeed fold around the Zn atom.

Previous studies reported that substitutions of Zn binding residues with Asn or Gln are compatible with Zn coordination, without compromising the enzymatic activity^27^,^28^. In the case of the Asn mutant of a Zn finger from the basic Krüppel-like factor, the mutation does affect the stability of structure, making it less compact and it has been suggested that this may provide an intriguing regulatory mechanism^27^. Similarly, it is possible that the asparagine mutation in the human Zn knuckle is important to produce a less stable but more versatile fold, able to adapt and modulate its structure according to the substrate bound. Zn knuckles are indeed known to be very adaptable scaffolds, with a mode of binding that depends on the nucleic acid target^21^,^29^.

We further decided to investigate the role of the additional conserved region upstream the Zn knuckle motif, including the segment with similarity to the C-terminus of Sld2 (Fig. 1). We cloned and expressed two fragments encompassing these regions, in both the human and *Xenopus* RecQ4 (constructs hUpZnK and xUpZnK) and carried out a detailed biochemical investigation with both DNA and RNA substrates. We found that the new constructs bind nucleic acid with a much higher affinity (Table 2, Fig. 3C,D) compared to the Zn knuckles alone. Again, there is a clear preference for RNA substrates over DNA. Thus the upstream region strongly enhances nucleic acid binding, for both the human and *Xenopus* proteins.

In agreement with the notion that the upstream region is a strong determinant of nucleic acid binding, mutations of Zn^2+^ ligands in the human construct only partially impair nucleic acid interactions (Fig. 4). The analysis of the mutations suggests that although the Zn knuckle has a weaker affinity, it works synergistically with the upstream region. In particular, mutants affecting the Zn ligands weaken the binding to ssDNA and forkDNA substrates. Consistently, the presence of EDTA impairs binding to DNA substrates. The effect of EDTA is stronger than what observed for the mutants, suggesting that it may have a more significant impact on the folding and/or stability of the domain. The Asn to Cys substitution (which reconstitute a canonical Zn binding site) does not improve binding to nucleic acid substrates. Indeed the mutation negatively affects binding to DNA substrates similarly to the disrupting mutations, suggesting that the human ligands are optimized for the function of the protein.

**Figure 5:**
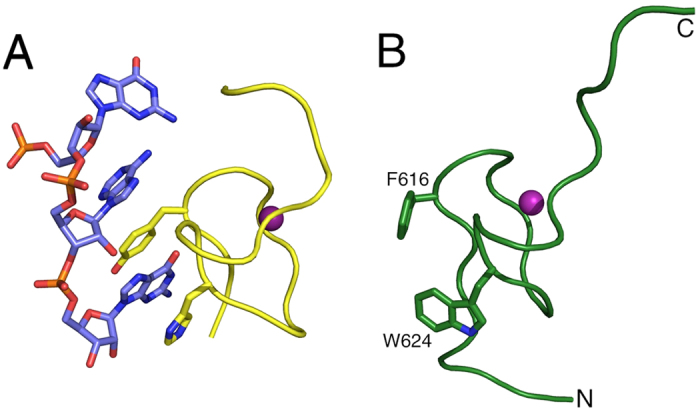
Putative mode of nucleic acid binding. (**A**) A detail of the Zn knuckle-RNA interactions of mouse Lin28 with let-7 microRNA^21^ (PDB code: 3TRZ), showing the critical role of aromatic residues stacking with the nucleic acid basis. (**B**) The *Xenopus* Zn knuckle is shown in the same orientation; for the sake of clarity, secondary structure elements are not depicted. The two conserved hydrophobic residues Phe616 and Trp624 are shown as ball-and-stick.

The structures of a number of Zn knuckles have been determined in the presence of nucleic acid substrates^20^,^21^,^29^,^30^,^31^,^32^. Although this fold is very versatile, the pattern that emerges is the presence of a nucleic acid binding platform mainly formed by exposed aromatic residues, making *π*-*π* stacking interactions with the bases. Indeed the RecQ4 Zn knuckle comprises two conserved aromatic residues (Phe616 and Trp624 in the *Xenopus* sequence) that are exposed and equivalent to the residues that sandwich the bases in the known complexes (Fig. 5).

Less easy is to predict the molecular details of the interactions with the upstream region, in the absence of structural information. Bioinformatic analysis predicts the presence of two short β-strands (in the region homologous to Sld2) followed by a long α-helix (Fig. 1C). Genetic studies in *S. cerevisiae* identify a positively charged residue (Lys438) at the C-terminus of Sld2 which is essential for binding to DNA^33^. In RecQ4 proteins, this residue is substituted by a conserved asparagine (Asn356 in the human orthologue), but a number of positively charged and aromatic residues occur on the same side of the predicted β-strand. The predicted helix, unique to RecQ4 sequences, is also very rich in aromatic and positively charged residues (Fig. 1C).

Two recent reports^23^,^24^ focus on the biochemical characterization of fragments of the N-terminal domain of human RecQ4: they both analyse various deletion mutants, but neither tackles the Zn knuckle. One of the papers^23^ describes a high affinity to Holliday junctions, mostly due to the region between residue 322 to 400 (which corresponds to the upstream region); although the authors do not attempt a quantitative analysis, the affinity reported for the Holliday junction seems comparable to the affinity we measure towards RNA substrates. It is difficult to compare our data with the result of the second paper^24^, which reports an affinity for G quadruplets, as the EMSAs do not show discrete bands, and indicates a high degree of aggregation, so that most of the protein-nucleic acid complexes are trapped into the wells.

At the moment, we can only speculate on the role of the Zn knuckle within the context of RecQ4. RecQ4 seems to have a dual role, with the Sld2-like N-terminal region essential for the initiation of DNA replication and the helicase domain likely to be involved in one or more DNA damage repair pathways. The fragment here characterized is located between these two domains and could potentially contribute to either one or the other cellular process. For example yeast Sld2 has been reported to bind to origin DNA^34^, and this binding could be enhanced by the Zn knuckle. At the same time it could act as an additional DNA binding domain within the helicase context.

The most intriguing finding is the ability of this region to bind RNA substrates, raising a number of questions as to the possible physiological significance of this observation. A growing role for RNAs is emerging in a large number of cellular processes. Non-coding RNAs have been shown to play roles in the initiation of eukaryotic DNA replication in different classes of organisms, such as Y RNAs in higher eukaryotes, G-quadruplex RNAs in the replication of the Epstein-Barr and its human host cells and 26T RNA in *Tetrahymena*^35^. R-loop formation seems to occur more frequently than previously thought and is a potential source of genome instability at the interface between transcription and replication^36^. Genomic integrity is also dependent on non-coding RNAs: not only they are involved in transcriptional/translational regulation of DNA repair factors, but there is evidence for a more direct role for ncRNAs generated at the site of the DNA damage in establishing repair foci^37^ and a template role for RNA in the repair mechanism has also been suggested^38^. The RNA binding capability we describe in this paper suggests that RecQ4 may be involved one or more of these processes.

## Methods

### Peptides synthesis

Peptides corresponding to the putative Zn knuckle domains of human and *Xenopus* RecQ4 (pep-hZnK, residue number 397 to 421, and pep-xZnK, residue number 609 to 633, Fig. 1) were synthesized using an Applied Biosystems Peptide Synthesizer. Purification of the peptides was performed using C18, 6micron (Waters) reverse phase column. The final product was lyophilized and characterized by analytical reverse phase HPLC, amino acid analysis, and laser desorption mass spectrometry (Peptide Facility, CRIBI, Biotechnology Centre, Padua).

### Cloning, expression and purification of RecQ4 Zn knuckle and mutants

Two fragments encompassing amino acids 394–427 and 607–638 of human and *Xenopus* RecQ4 Zn knuckles (hZnK, 32.7 kDa and xZnK, 32.5 kDa respectively, Fig. 1) were PCR-amplified and cloned into a pETM-30 vector (EMBL) through a restriction-free (RF) cloning method^39^. The vector is designed to express a fusion protein including a 6His-GST tag upstream the target sequence.

Two additional constructs were designed to include the conserved upstream region. A fragment corresponding to the *Xenopus* Zn knuckle and the upstream region (residues 555–638, xUpZnK, 38.4 kDa, Fig. 1) was subcloned in a pETM-30 vector with the RF cloning method. A fragment comprising the Zn knuckle and upstream region of human RecQ4 (residues 335–427, hUpZnK, 39.7 kDa, Fig. 1), was subcloned into pETM-30 using restriction enzymes *NcoI* and *BamHI*. Site directed mutagenesis (Invitrogen) was performed on this vector and critical residues were mutated (Mutant m1: Cys403Ala/Asn406Ala; Mutant m2: His411Ala/Cys416Ala; Mutant m3: Asn406Cys).

GST-tagged wild type and mutant proteins were expressed in Rosetta 2 cells in Terrific Broth (TB, Sigma) at 18 °C overnight, following induction with 0.2 mM IPTG in the presence of 0.1 mM ZnSO_4_. Cells were lysed by sonication in 50 mM Hepes pH 7.5, 0.5 M NaCl, 1 mM TCEP, 5% glycerol, 10 mM Imidazole, 1 Tablet Complete Protease Inhibitor (Roche) and DnaseI (Sigma). Proteins were purified by Nickel-affinity chromatography, washed with 2 M NaCl to eliminate DNA contamination and run on a size exclusion chromatography (Superdex-200) in 250 mM NaCl, 20 mM Tris pH 7.5, 5% glycerol and 5 mM β-mercaptoethanol. The fusion tag was not cleaved from the proteins, due to the difficulties of purification of such small and flexible untagged fragments.

### Oligonucleotides preparation

All oligonucleotides were chemically synthesized and purified by reverse-phase high pressure liquid chromatography (Sigma-Aldrich, Suffolk, UK). Each oligonucleotide was then resuspended in Tris–EDTA (TE) buffer (10 mM Tris–HCl, pH 7.5, 1 mM EDTA, pH 8.0), supplemented with RNase inhibitor (NEB) in case of RNA substrates. Oligonucleotide sequences used in this work are reported in Supplementary Table S1 online. The forked substrates comprised 22 base paired and 15 unpaired and were obtained by annealing oligonucleotides A1 and A2 (DNA fork) and A3 and A4 (RNA fork).

For radioactive EMSA, a single oligonucleotide was 5′-end-labeled with [γ-^32^P] ATP using T4 polynucleotide kinase. The kinase reaction was performed in PNK buffer (70 mM Tris–HCl, pH 7.6, 10  mM MgCl_2_, 5 mM dithiothreitol) at 37 °C for 45 min.For fork and double-stranded probes, the [γ-^32^P] ATP-labeled oligonucleotides were then annealed to a 1.6-fold excess of the unlabeled complementary strands in annealing buffer (10 mM Tris–HCl, pH 7.5, 50 mM NaCl) by heating at 95 °C for 8 min and then cooling slowly to room temperature. The purification of the duplex substrates was performed using Micro Bio-Spin columns (Bio-Rad).

### CD spectroscopy

Circular dichroism (CD) spectra of both synthetic peptides (pep-hZnK and pep-xZnK) were recorded at 25 °C on a Jasco J-810 Spetropolarimeter at wavelength 190–260 nm, in 0.1 cm quartz cells, band width 1 nm, response 1 sec, data pitch 0.1 nm and scanning speed 20 nm/min. Each peptide was dissolved at 20 μM in 50 mM phosphate buffer pH 7.4, 0.1 mM TCEP. Where indicated, Zn^2+^, DNA or RNA were added in an equimolar ratio. Three scans were done for each sample and averaged. Every spectrum was corrected for the buffer and the RNA/DNA contribution when relevant.

### Electrophoretic Mobility Shift Assays

The experiments were performed by incubating increasing concentrations of purified recombinant wild type and mutant RecQ4 fragments (fused to the GST tag) with [γ-^32^P] labelled oligonucleotides at a final concentration of 10 nM in a 20 μl reaction mixture containing 20 mM Tris-HCl pH 7.5, 2 mM MgCl_2_, 50 mM NaCl, 5% glycerol, 1 mM DTT, 5 mM ZnCl_2_ and 0.1 mg/ml BSA. EMSA in the presence of EDTA were carried out in the same conditions at a fixed protein concentration of 500 nM, and increasing amounts of EDTA (0–50 mM). After incubation for 30 min at room temperature, the reaction products were separated on a 6% non denaturing polyacrylamide gel run at 4 °C in TBE buffer. Labeled nucleic acid fragments were detected by autoradiography (Cyclon, GE Healthcare) and quantification of protein-nucleic acid complexes was performed with ImageQuant image analysis software (GE Healthcare). The apparent equilibrium dissociation constants (*K*_D_) were determined using the single-site specific binding model as implemented in Prism (GraphPad software) from the mean of three independent experiments.

### NMR spectroscopy and resonance assignments

NMR experiments were performed at 298 K on a Bruker Avance 600 MHz spectrometer equipped with inverse triple resonance cryoprobe (TCI) and pulsed field gradients. Data were processed with Topspin 2.0 (Bruker) and analyzed using CCPNmr 2.1.5^40^. 1D ^1^H spectra were acquired on 0.3 mM human pep-hZnK and 0.3 mM *Xenopus* pep-xZnK samples in 20 mM NaH_2_PO_4_/Na_2_HPO_4_ pH 6.3, 150 mM NaCl, 4 mM DTT, 0.3 mM DSS, 10% (v/v) D_2_O, with or without 0.35 mM ZnCl_2_. 1D ^1^H spectra were also acquired on 0.36 mM pep-hZnK sample in 50 mM HEPES pH 6.8, 150 mM NaCl, 4 mM DTT, 0.3 mM DSS, 10% D_2_O, with or without 0.35 mM ZnCl_2_, and 0.09 mM pep-hZnK in 50 mM HEPES pH 6.8, 150 mM NaCl, 4 mM DTT, 0.09 mM ZnCl_2_, 0.3 mM DSS, 10% D_2_O with or without 0.09 mM ssDNA (A1, Table ST1). The NOESY experiments were performed using the Bruker library pulse sequence noesyesgpph, which employs excitation sculpting with gradients to suppress water^41^.

^1^H, resonances of pep-xZnK were assigned analysing ^1^H-TOCSY (mixing time 60 ms) and ^1^H-NOESY (mixing time 100, 150 and 200 ms) spectra. ^15^N, ^13^Cα and Cβ resonances were also assigned analysing respectively 2D ^1^H-^15^N HSQC and ^1^H-^13^C HSQC spectra performed exploiting the natural abundance of the ^15^N isotope and ^13^C isotopes. For these experiments the sample concentration was 1 mM in in 20 mM NaH_2_PO_4_/Na_2_HPO_4_ pH 6.3, 150 mM NaCl, 4 mM DTT, 1.25 mM ZnCl_2_, 0.3 mM DSS, 10% (v/v) D_2_O.

### Structure calculation

The solution structure of the *Xenopus* Zn knuckle (pep-xZnK) was calculated using ARIA 2.3.1^42^ in combination with CNS^43^, based on the experimentally derived restraints (Table 1). In particular, proton–proton distances were obtained from a 2D ^1^H-^1^H NOESY spectrum (mixing time 200 ms). Φ/Ψ restraints were obtained from backbone chemical shifts using TALOS+^44^. Hydrogen bond restraints were defined from slow-exchanging amide protons identified after exchange of the H_2_O buffer to D_2_O. The 2D ^1^H-^1^H NOESY spectrum was manually assigned and calibrated by ARIA. Eight ARIA iterations were performed, 100 structures were computed in the last iteration and ARIA default water refinement was performed on the 15 best structures from the final round. Initial structures were calculated without Zn ion restraints to verify the position and the geometry of the metal ion ligands, so that the residues involved in Zn binding could be identified in an unbiased manner. Several NOEs were observed between metal coordinating residues, clearly revealing the tetrahedral coordination of the ligands around the Zn ion. The tautomeric state of His623 (Nδ1 protonated) was deduced based on the pattern of NOEs established by the aromatic protons of His623 (Supplementary Fig. S4 online). Once metal ligands were unequivocally identified, the geometry of the Zn^2+^ coordination was constrained in the final ARIA calculations via covalent bonds and angles in the CNS parameters; the tetrahedral angles and distances for Zn^2+^ coordinating atoms (Zn-S^-^ Zn-Nε) were maintained also after water refinement. Structural quality was assessed using Procheck-NMR^45^ and molecular images were generated by PyMOL (http://pymol.org/). The family of the 15 lowest energy structures for *Xenopus* RecQ4 Zn knuckle has been deposited in the PDB (Protein Data Bank) with the accession code 2MPJ. Chemical shift and restraints used in the structure calculations have been deposited in BioMagResBank (19986 code).

## Additional Information

**How to cite this article**: Marino, F. *et al.* Structural and biochemical characterization of an RNA/DNA binding motif in the N-terminal domain of RecQ4 helicases. *Sci. Rep.* **6**, 21501; doi: 10.1038/srep21501 (2016).

## Supplementary Material

Supplementary Information

## Figures and Tables

**Table 1:** Structural statistics of *Xenopus* Zn knuckle (pep-xZnK).

	<SA > pep-xZn^a^
Restraints information	
NOE restraints^b^	
all	167
intra [0]	87
sequential [1]	49
short [2,3]	12
medium [4,5]	10
long [>5]	9
Dihedral angles (phi and psi)^c^	14
Hydrogen bonds^d^	2
Zn^2+^ coordination	4
RMSD (Å)^e^	
Ordered heavy atoms	0.564 ± 0.119
Ordered backbone atoms (N, Ca, C’)	0.270 ± 0.096
Average RMSD from experimental restraints	
All experimental distances restraints (Å)	0.137 ± 0.010
All dihedral angle restraints (°)	0.542 ± 0.028
Ramachandran quality parameters (%)^e^	
residues in most favoured regions	83,3
residues in allowed regions	16,7
residues in additional allowed regions	0,0
residues in disallowed regions	0,0

^a^Simulated annealing, statistics refer to the ensemble of 15 structures with the lowest energy.

^b^No distance restraint in any of the ensemble structures was violated more than 0.5 Å.

^c^No dihedral angle restraint in any of the ensemble structures was violated more than 5°.

^d^The following hydrogen bond restraints have been used for the calculations: Gly622NH-Asp613CO; Cys615NH-Gly620CO (upper limit distance 2.67 Å, lower limit distance 1.7 Å).

^e^Statistics are given for residues T614-C628.

**Table 2:** Apparent equilibrium dissociation constants (*K*_D_, expressed in nM) for hUpZnK and xUpZnK with various nucleic acid substrates.

	ssDNA	ssRNA	ForkDNA	ForkRNA
hUpZnK	185 ± 33	11 ± 0.7	42 ± 2.8	13 ± 1.1
xUpZnK	25 ± 2.0	7.3 ± 0.4	14 ± 2.3	5.4 ± 0.4
