# Supplementary material for: Structural and biochemical characterization of an RNA/DNA binding motif in the N-terminal domain of RecQ4 helicases
Source: Sci Rep. 2016 Feb 18;6:21501. doi: 10.1038/srep21501 (PMC4757822; doi:10.1038/srep21501)
Supplement: Supplementary Information [file srep21501-s1.pdf]

Supplementary Information

Structural and biochemical characterization of an RNA/DNA binding motif in the N-terminal domain of RecQ4 helicases.

Francesca Marino, Aditya Mojumdar, Chiara Zucchelli, Amit Bhardwaj, Emanuele Buratti, Giovanna Musco, Alessandro Vindigni and Silvia Onesti

Supplementary Table S1.

Oligonucleotides used in this study (DNA, upper case; RNA, lower case).

Name	Sequence 5'-3'
A1	GTAGTGCATGTACACCACACTCTTTTTTTTTTTTTTT
A2	TTTTTTTTTTTTTTTGAGTGTGGTGTACATGCACTAC
A3	guagugcauguacaccacacucuuuuuuuuuuuuuuu
A4	uuuuuuuuuuuuuuugagugugguguacaugcacuac

Supplementary Figure S1

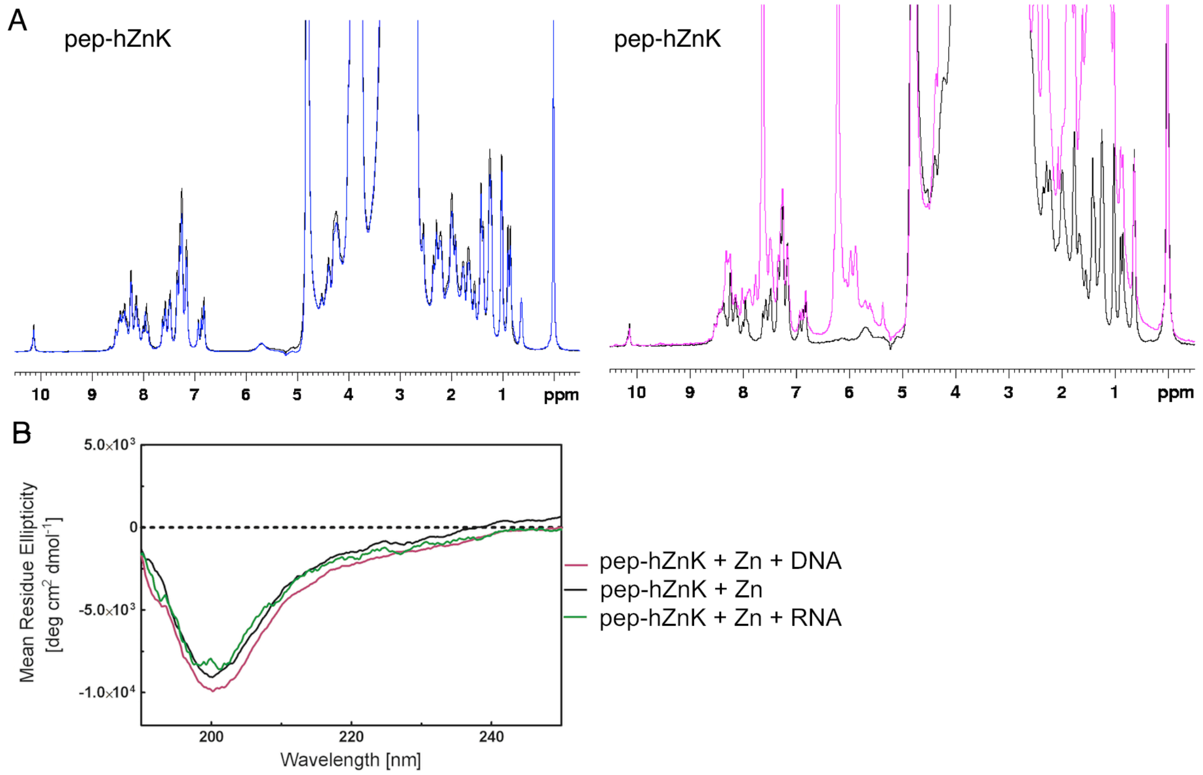

Figure S1. Additional spectroscopic studies of pep-hZnK

(**A**) Left: NMR 1D ^{1}H spectrum of pep-hZnK in HEPES buffer without (black) and with (blue) zinc. Right: NMR 1D ^{1}H spectrum of pep-hZnK in presence (pink) or absence (black) of Zn^{2+} and ssDNA. (**B**) CD spectra of pep-hZnK: no secondary structure appears after the addition of Zn^{2+} (black) or Zn^{2+} plus ssDNA (pink) or ssRNA (green).

Supplementary Figure S2

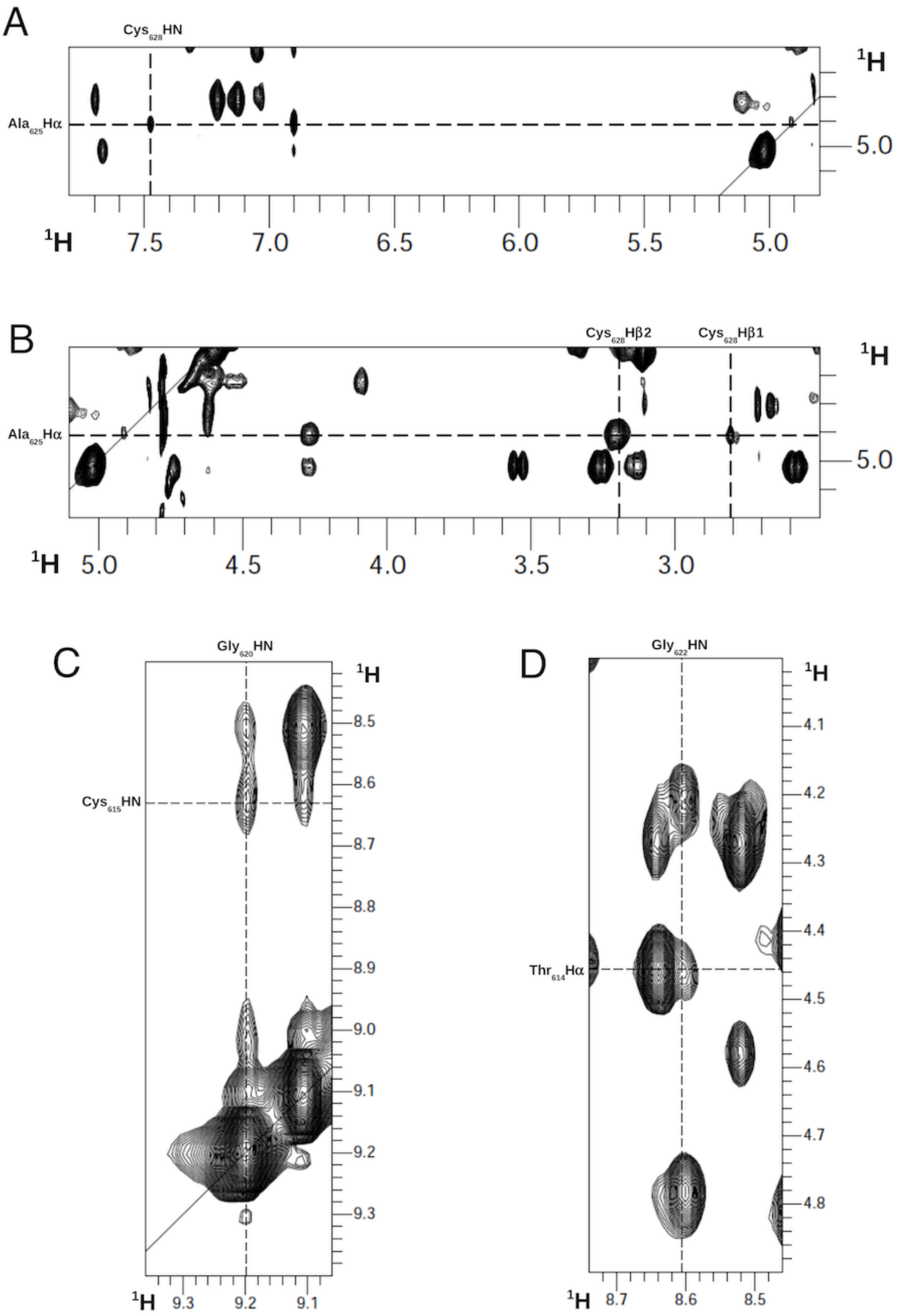

Figure S2. 1H-2D NOESY spectrum of pep-xZnK.

Selected regions of the 1H-2D NOESY spectrum showing the NOE cross-peaks between (**A**) Cys628HN and Ala625HA, (**B**) Ala625Hα/Cys628Hβ1/Hβ, (**C**) Gly620HN/Cys615HN, (**D**) Gly622HN/Thr614Hα

Supplementary Figure S3

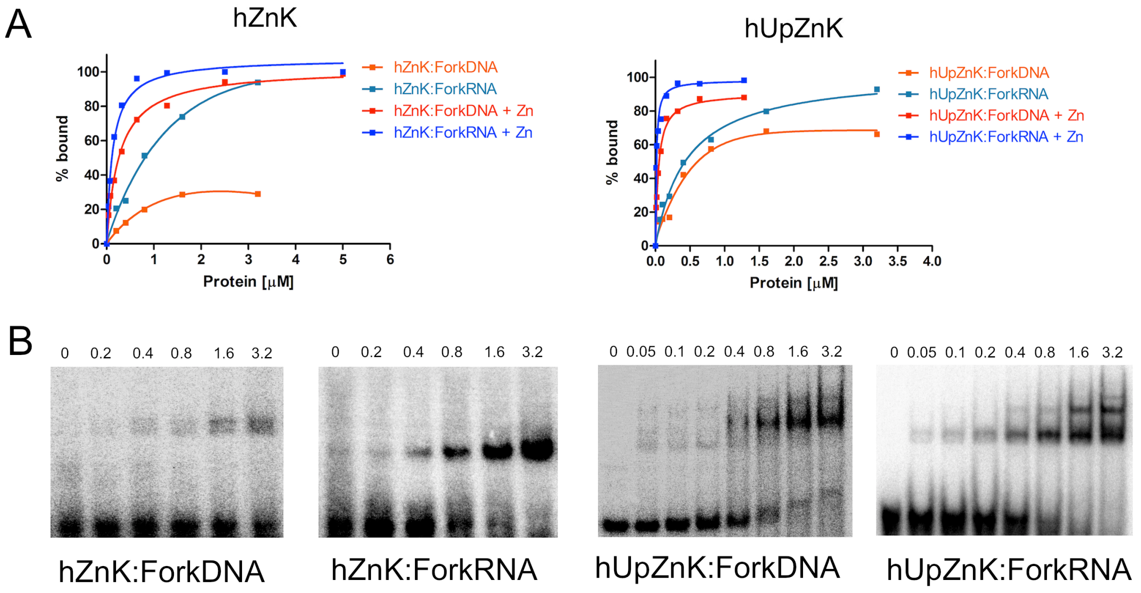

Figure S3. The effect of the presence of Zn^{2+} ions in the binding buffer on the interaction between the human RecQ4 fragments and forked substrates.

(**A**) The plots compare the binding of forked DNA and RNA substrates in the presence of 5mM $ZnCl_2$ (red and blue curves respectively), as reported in Figure 3, and the binding in the absence of Zn^{2+} ions in the binding buffer (orange and teal curves, respectively). (**B**) Example of gel shifts assays: the experiments were carried out with increasing concentrations of His-GST-tagged recombinant proteins (0-3.2 μM). Each experiment was repeated at least three times to plot the binding curves. Errors were very small: for the sake of clarity error bars are not shown on the plots. The proteins do bind nucleic acids also in the absence of Zn^{2+} ions, but the presence of Zn^{2+} in the buffer significantly improves binding to both DNA and RNA substrates, indicating that Zn^{2+} plays a relevant role in the interaction, and suggesting that the folding of the Zn knuckle is indeed important for the interaction.

Supplementary Figure S4

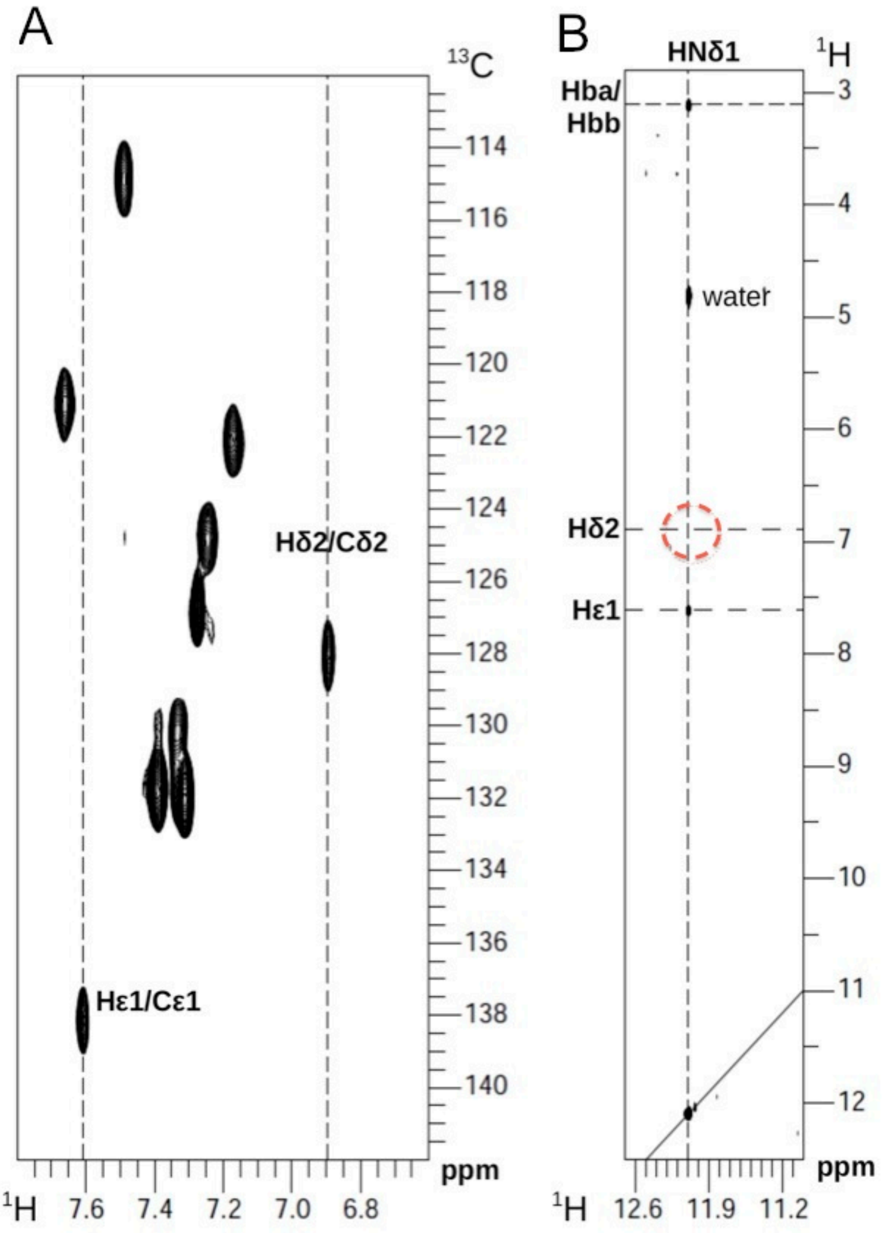

Figure S4.

(A) 13C-HSQC experiment (natural abundance) used to assign the aromatic resonances (1H and 13C) of His623. **(B)** Zoom on the NOESY spectrum (tmix=100ms) showing the NOEs between HNδ1 and i. its own beta-protons (Hba and Hbb are isochronous) and ii. Hε2. The presence of these NOEs and the absence of the NOE (red dotted circle) with Hδ2 support Zn2+ coordination via Nε2.
